# Prevalence, correlates, and mental health burden associated with homelessness in U.S. military veterans

**DOI:** 10.1017/S0033291722000617

**Published:** 2023-07

**Authors:** Brandon Nichter, Jack Tsai, Robert H. Pietrzak

**Affiliations:** 1 Department of Psychiatry, Yale School of Medicine, New Haven, CT, USA; 2 U.S. Department of Veterans Affairs, National Center on Homelessness Among Veterans, Tampa, FL, USA; 3 University of Texas Health Science Center at Houston, Houston, TX, USA; 4 U.S. Department of Veterans Affairs, National Center for Posttraumatic Stress Disorder, VA Connecticut Healthcare System, West Haven, CT, USA; 5 Department of Social and Behavioral Sciences, Yale School of Public Health, New Haven, CT, USA

**Keywords:** ACES, homelessness, military, suicide, trauma, veterans

## Abstract

**Background:**

Homelessness is a major public health problem among U.S. military veterans. However, contemporary, population-based data on the prevalence, correlates, and mental health burden of homelessness among veterans are lacking.

**Methods:**

Data were analyzed from the 2019–2020 National Health and Resilience in Veterans Study, a nationally representative survey of veterans (*n* = 4069). Analyses examined the prevalence and correlates of homelessness, as well as the independent associations between homelessness and current probable psychiatric conditions, suicidality, and functioning.

**Results:**

The lifetime prevalence of homelessness was 10.2% (95% confidence interval 9.3–11.2). More than 8-of-10 veterans reported experiencing their first episode of homelessness following military service, with a mean of 10.6 years post-discharge until onset (s.d. = 12.6). Adverse childhood experiences (ACEs), cumulative trauma burden, current household income, younger age, and drug use disorder emerged as the strongest correlates of homelessness (49% of total explained variance). Veterans with a history of homelessness had elevated odds of lifetime suicide attempt, attempting suicide two or more times, and past-year suicide ideation [odd ratios (ORs) 1.3–3.1]. They also had higher rates of current probable posttraumatic stress disorder, major depressive, generalized anxiety, and drug use disorders (ORs 1.7–2.4); and scored lower on measures of mental, physical, cognitive, psychosocial functioning (*d* = 0.11–0.15).

**Conclusions:**

One in ten U.S. veterans has experienced homelessness, and these veterans represent a subpopulation at substantially heightened risk for poor mental health and suicide. ACEs were the strongest factor associated with homelessness, thus underscoring the importance of targeting early childhood adversities and their mental health consequences in prevention efforts for homelessness in this population.

Homelessness is a major public health problem in the United States. In 2020, the number of people experiencing homelessness in the U.S. was estimated to be approximately 600 000, although the true figure is likely much higher [Department of Housing and Urban Development (HUD), 2021a]. Military veterans comprise a substantial proportion of the U.S. homeless population and they represent a group at elevated risk of experiencing homelessness [Department of Veterans Affairs (VA), 2021]. Although the U.S. has seen substantial progress in reducing homelessness among veterans over the past two decades, with the rate of veteran homelessness decreasing by nearly half since 2009, it is estimated that on a single night in 2020, at least 37 000 veterans were experiencing homelessness (HUD, 2021b). This estimate was made prior to the onset of the coronavirus disease 2019 (COVID-19) pandemic, and there may be increased rates of housing instability and homelessness in the aftermath of the pandemic (Tsai, Szymkowiak, & Pietrzak, 2020). Preventing and ending veteran homelessness is currently a major federal priority for the U.S. Department of Housing and Urban Development and the VA (HUD, 2021c), given that a substantial body of research has found that homelessness is associated with a myriad of physical (e.g. cardiovascular disease, hepatitis) and psychiatric [e.g. posttraumatic stress disorder (PTSD), substance use disorders] conditions, as well as all-cause mortality (Brenner et al., 2017; Tsai, 2018; Weber, Lee, & Martsolf, 2017). Such findings underscore the importance of characterizing the prevalence, correlates, and burden of homelessness in contemporaneous, population-based samples of U.S. military veterans who have experienced homelessness.

A large body of prior epidemiologic studies has characterized the prevalence and correlates associated with homelessness among veterans. Taken together, this body of evidence suggests that the lifetime prevalence of veterans who have experienced homelessness ranges between 4.2% and 10.0%, depending on a variety of methodologic factors, such as the data collection method (e.g. self-report survey, VA administrative health records; Edens, Kasprow, Tsai, & Rosenheck, 2011; Peterson et al., 2015; Tsai, 2018; Tsai & Cao, 2019; Tsai, Link, Rosenheck, & Pietrzak, 2016). With respect to sociodemographic characteristics, veterans who report a history of lifetime homelessness tend to be male, less highly educated, unpartnered/divorced, non-White, lower income, and unemployed (Tsai et al., 2016; Tsai & Rosenheck, 2015). With respect to military service characteristics, veterans who report a history of homelessness are more likely to have enlisted into the military (*v.* drafted), received an other-than-honorable or dishonorable discharge, and served significantly less time in the military relative to those without such histories (Metraux, Clegg, Daigh, Culhane, & Kane, 2013; Tsai et al., 2016). Regarding psychiatric characteristics, veterans who report lifetime homelessness are more likely to have PTSD, major depressive, alcohol, and drug use disorders, as well as serious mental illness (e.g. bipolar spectrum, psychotic disorder; Brenner et al., 2017; Metraux et al., 2013). In terms of psychosocial characteristics, veterans with histories of homelessness report more adverse childhood experiences (ACEs; Spinola, Hoff, & Tsai, 2021), poorer physical functioning (Tsai et al. 2016), greater disability (Tsai & Rosenheck, 2015), and higher rates of suicidal ideation and attempts (Holliday et al., 2021; Schinka, Schinka, Casey, Kasprow, & Bossarte, 2012).

Although several previous studies have examined the prevalence and correlates of homelessness among veterans in the United States, extant literature in this area is limited in three notable ways. First, a significant proportion of prior studies have utilized Veterans Health Administration (VHA) records to characterize the prevalence of veterans who have experienced homelessness, yet the vast majority (83.1%) of U.S. veterans do not utilize VA as their primary source of healthcare (Meffert et al., 2019). This limitation is important, as recent evidence suggests VHA-users and non-users may differ with regard to sociodemographic and clinic characteristics, such as being more likely to be younger, female, non-White, lower income, and having a poorer physical and mental health (Meffert et al., 2019). Second, while numerous prior studies have examined the role of sociodemographic, military, and psychiatric characteristics of veterans with a history of homelessness, scarce research has examined whether other psychological characteristics such as personality traits (e.g. emotional stability) and psychosocial features such as attachment style may be linked to homelessness. Third, although previous research has established a link between homelessness and suicidality among veterans, few population-based studies have examined whether a history of homelessness is independently associated with suicidal thoughts and behaviors, after adjustment for sociodemographic, military, trauma, psychiatric, and substance use disorder characteristics. Such data are critical to informing the population-based burden of homelessness among veterans in the United States; developing targeted prevention and intervention strategies; and guiding resource allocation.

The current study sought to address these gaps in the literature by analyzing data from the 2019–2020 National Health and Resilience in Veterans Study (NHRVS), a nationally representative survey of more than 4000 U.S. veterans, to evaluate the following three aims: (a) estimate the lifetime prevalence of homelessness in the U.S. veteran population; (b) identify sociodemographic, military, psychiatric, trauma, and psychosocial risk variables most strongly associated with a history of homelessness; and (c) examine the independent association between lifetime history of homelessness and current probable psychiatric disorders, current functioning, and lifetime and current suicidal thoughts and behaviors.

## Method

Data were analyzed from the 2019–2020 NHRVS, a nationally representative study of 4069 U.S. veterans. The mean age of the sample was 62.2 (s.d. = 15.7 range = 22–99; 90.2% male). Participants were 78.0% non-Hispanic Caucasian, 11.2% non-Hispanic Black, 6.6% Hispanic, and 4.2% other, mixed race. A total of 37.1% served 3 or fewer years, 42.1% served 4–9 years, and 20.8% served 10 or more years in the military; a total of 47.0% served in the Army, 20.2% Navy, 18.7% Air Force, 5.8% Marines, and 8.3% National Guard, Reserves, or Coast Guard, and 35.0% were combat veterans.

The sampling methodology of the NHRVS has been described elsewhere (Nichter et al., 2021a, 2021b). Briefly, veterans completed an anonymous, web-based survey. The NHRVS sample was drawn from KnowledgePanel^®^, a panel of more than 50 000 households maintained by Ipsos, a research firm. KnowledgePanel^®^ is a probability-based survey panel of a representative sample of U.S. adults that covers approximately 98% of U.S. households. Panel members are recruited through national random samples, originally by telephone and now almost entirely by postal mail. KnowledgePanel^®^ recruitment uses sampling frames that include both listed and unlisted telephone numbers, telephone and non-telephone households, and cell-phone-only households, as well as households without Internet access. In the recruitment process, KnowledgePanel employed an initial screening question that confirmed veteran status (‘Have you ever served on active duty in the U.S. Armed Forces, Military Reserves, or National Guard?’). To permit generalizability of results to the entire U.S. veteran population, Ipsos computed post-stratification weights using the following benchmark distributions of U.S. veterans from the most recent (August 2019) Current Veteran Population Supplemental Survey of the Census Bureau's American Community Survey: age, sex, race/ethnicity, metropolitan status, education, household income, a branch of service, and years in service. An iterative proportional fitting (raking) procedure was used to produce the final post-stratification weights. Missing data (<3%), which were missing completely at random as per Little's Missing Completely at Random (MCAR) test, were multiply imputed using chained equations. Raw unweighted frequencies are reported while poststratification weights were applied when computing prevalence and inferential statistics to allow for generalizability to the U.S. veteran population. Participants provided informed consent and the study was approved by the Human Subjects Subcommittee of the VA Connecticut Healthcare System.

### Assessments

Study measures are described in Table 1.

**Table 1.** Study measures

History of homelessness	Lifetime homelessness was assessed using a dichotomous item (yes/no), ‘In your entire adult life, have you ever been homeless (i.e. stayed in a shelter, transitional housing, outdoors, or some other unstable housing situation)?’
Psychiatric factors	
Lifetime and current posttraumatic stress disorder (PTSD)	Score of ⩾33 on PTSD Checklist for DSM-5 (Weathers et al., 2013b; *α* = 0.95), modified to assess both lifetime and past-month ratings of PTSD symptoms in relation to ‘worst’ Criterion A trauma on the Life-Events Checklist-5 (LEC-5; Weathers et al., 2013a).
Lifetime major depressive disorder (MDD)	Lifetime MDD was assessed using the modified self-report version of the MDD module from the DSM-5 version of the Mini Neuropsychiatric Interview (Sheehan, 2016)
Lifetime alcohol use disorder	Lifetime alcohol use disorder was assessed using a modified version of AUD module from the DSM-5 version of the Mini Neuropsychiatric Interview (Sheehan, 2016)
Lifetime drug use disorder	Lifetime drug use disorder was assessed using a modified self-report version of DUD module from the DSM-5 version of the Mini Neuropsychiatric Interview (Sheehan, 2016)
Lifetime nicotine use disorder	Lifetime nicotine use disorder was defined as a score ⩾5 on the Fagerström Test for Nicotine Dependence (FTND) scale was considered a positive screen (Heatherton, Kozlowski, Frecker, & Fagerstrom, 1991)
Current major depressive disorder	Score ⩾3 on the depression items of the Patient Health Questionnaire-4 (Löwe et al., 2010)
Current generalized anxiety disorder	Score ⩾3 on the anxiety items of the Patient Health Questionnaire-4 (Löwe et al., 2010)
Current alcohol use disorder	Score ⩾8 on the Alcohol Use Disorders Identification Test (Saunders, Aasland, Babor, De la Fuente, & Grant, 1993)
Current drug use disorder	Score ⩾7 on the Screen of Drug Use (Tiet et al., 2015), which asked ‘How many days in the past 12 months have you used drugs other than alcohol?’ or score of ⩾2 to the question: ‘How many days in the past 12 months have you used drugs more than you meant to?’
Suicidal behaviors	
Past-year suicide ideation	Past-year suicide ideation was assessed via positive endorsement of Question 2 of the Suicide Behaviors Questionnaire-Revised (SBQ-R; Osman et al., 2001): ‘How often have you thought about killing yourself in the past year?’ Response options range from Rarely (1 time) to Very Often (5 + times).
Lifetime suicide attempt	Lifetime suicide attempt was assessed via positive endorsement of either of two items on Question 1 of the SBQ-R: ‘I have attempted to kill myself, but did not want to die’ and ‘I have attempted to kill myself, and really hoped to die.’ An additional question assessed number of lifetime attempts.
Clinical and health characteristics	
Adverse childhood experiences	Adverse Childhood Experiences Questionnaire total score (Felitti et al., 1998)
Military sexual trauma	Endorsement on either or both items from the VHA MST screen, which asks, ‘When you were in the military, did you ever receive unwanted, threatening, or repeated sexual attention?’ and ‘When you were in the military, did you have sexual contact against your will or when you were unable to say no?’
Number of lifetime traumas	Life Events Checklist for DSM-5 total score (Weathers et al., 2013a)
Physical health problems	Sum of number of medical conditions: ‘Has a doctor or healthcare professional ever told you that you have any of the following medical conditions?’ (e.g. arthritis, cancer, concussion, traumatic brain injury).
Personality characteristics	Score on the agreeableness, conscientiousness, emotional stability, and openness to experience subscales of the Ten-Item Personality Inventory (Gosling, Rentfrow, & Swann, 2003).
Protective psychosocial characteristics	
Secure attachment	Endorsement of secure attachment (response a) to the following question: ‘Please select the statement below that best describes your feelings and attitudes in relationships (Hazan and Shaver, 1990): (a) feeling that it is easy to get close to others and feeling comfortable with them (secure); (b) feeling uncomfortable being close to others (avoidant); or (c) feeling that others are reluctant to get close (anxious/ambivalent).
Perceived resilience	Score on Connor–Davidson Resilience Scale-10 (Campbell-Sills, & Stein, 2007)
Dispositional optimism	Score on single-item measure of optimism from Life Orientation Test-Revised (Scheier, Carver, & Bridges, 1994): ‘In uncertain times, I usually expect the best;’ Response options: 1 = strongly disagree to 7 = strongly agree
Functioning	
Mental health functioning	Medical Outcomes Study Short Form 8 Health Survey score (Stewart, & Ware,, 1992), which measures current role limitations caused by emotional problems, vitality, social functioning, and mental health. Lower scores indicate poorer functioning.
Physical health functioning	Medical Outcomes Study Short Form 8 Health Survey score (Stewart, and Ware,, 1992), which measures current overall physical functioning, physical role functioning, and general health. Lower scores indicate poorer functioning.
Cognitive functioning	Medical Outcomes Study Cognitive Functioning Scale score (Ware, Kosinski, Dewey, & Gandek, 2001), which measures several less severe day-to-day problems in six areas of current cognitive functioning. Lower scores indicate poorer functioning.
Psychosocial difficulties	Score on the Brief Inventory of Psychosocial Functioning (Kleiman et al., 2020)

### Data analysis

Data analyses proceeded in six steps. First, descriptive statistics were computed to estimate the lifetime prevalence of veterans with a history of homelessness. Second, sociodemographic, military, trauma, psychiatric, and psychosocial variables were compared by a history of homelessness using a series of univariate analyses of variance (ANOVAs) for continuous variables and chi-square analyses for categorical variables. Third, a series of multivariable logistic regressions were conducted to examine the independent associations between history of homelessness and current probable psychiatric comorbidities, adjusting for sociodemographic, military, and trauma-related characteristics. Fourth, a series of multivariable logistic regressions were conducted to examine independent associations between history of homelessness and suicide-related variables, adjusting for sociodemographic, military, and trauma-related characteristics, as well as lifetime major depressive, PTSD, and alcohol, drug, and nicotine use disorders. Fifth, multivariate ANOVA was conducted to compare scores on measures of functioning between veterans with and without a history of homelessness, adjusting for sociodemographic, military, and trauma characteristics, as well as the aforementioned psychiatric variables. Sixth, to determine the relative contribution of each sociodemographic, military, psychiatric, and clinical characteristic (Table 1) to the model explained variance (*R*^2^), a relative importance analysis (Tonidandel & LeBreton, 2011) was conducted using the relaimpo R statistical package. This analysis partitioned the explained variance in the history of homelessness that was explained by each significant variable while accounting for intercorrelations among these variables.

## Results

The weighted lifetime prevalence of homelessness in the sample was 10.2% [*n* = 343; 95% confidence interval (CI) 9.3–11.2]. Among veterans endorsing a history of homelessness, 82.4% (*n* = 259) reported that their first episode of being homeless occurred following military service. The mean number of years after service that veterans experienced homelessness was 10.6 (s.d. = 12.6); median = 4.0 years (interquartile range = 19.0). The mean number of months homeless among veterans reporting a history of homelessness was 15.8 (s.d. = 29.7); median = 6 months (interquartile range = 9.4). Approximately one-fourth (23.3%, *n* = 67) of veterans reported using VA healthcare or homelessness services while homeless.

Table 2 shows sociodemographic, military, clinical, psychiatric, and psychosocial characteristics of veterans with and without a history of homelessness. With respect to sociodemographic characteristics, relative to veterans without a history of homeless, those with a history of homelessness were younger, more likely to be female, racial/ethnic minorities, unmarried/partnered, have a current annual household income of <$ 60 000, and to have less than a college degree-level of education. With respect to military characteristics, veterans with a history of homelessness were more likely to have enlisted into the military, served in the Army, served 3 years or less in the military, and, among combat veterans, to have served in the conflicts of Iraq/Afghanistan or World War II. With respect to clinical characteristics, veterans with a history of homelessness were more likely to screen positive for lifetime probable depression, PTSD, alcohol, nicotine, and drug use disorders, as well as military sexual trauma and concussion/traumatic brain injury. They also reported a greater number of lifetime traumas, ACEs, and current medical conditions. With respect to personality characteristics, veterans with a history of homelessness reported lower levels of emotional stability, agreeableness, conscientiousness, and extraversion. With respect to protective psychosocial characteristics, veterans with a history of homelessness scored lower on measures of perceived resilience and dispositional optimism, and were less likely to report having a secure attachment style.

**Table 2.** Characteristics of U.S. military veterans by history of homelessness

	No history of homelessness *N* = 3708 weighted 89.8% Weighted mean (s.d.) or *N* (weighted %)	History of homelessness *N* = 343 weighted 10.2% Weighted mean (s.d.) or *N* (weighted %)	Test of difference	*p* value
Sociodemographic characteristics				
Age	63.2 (15.4)	52.9 (15.0)	12.95	<0.001
Male sex	3284 (91.0%)	265 (82.9%)	27.94	<0.001
Race/ethnicity			65.10	<0.001
White	3086 (79.8%)	218 (62.9%)		
Black	240 (10.1%)	55 (20.5%)		
Hispanic	268 (6.3%)	37 (9.4%)		
Other	114 (3.8%)	33 (7.2%)		
Education			44.28	<0.001
Less than high school	26 (1.5%)	3 (2.4%)		
High school graduate	468 (28.4%)	51 (33.2%)		
Some college	1486 (35.7%)	196 (45.9%)		
Bachelors or higher	1728 (34.4%)	93 (18.5%)		
Married/partnered	2703 (74.9%)	170 (50.4%)	112.41	<0.001
Annual household income <$60 K	1486 (38.9%)	219 (63.9%)	95.22	<0.001
*Military characteristics*				
Enlistment status			56.88	<0.001
Enlisted	2797 (78.1%)	316 (93.7%)		
Commissioned	461 (10.2%)	18 (2.2%)		
Drafted	445 (11.7%)	9 (4.1%)		
Years of military service			14.06	<0.001
3 or less	1358 (36.2%)	146 (44.6%)		
4–9	1525 (42.4%)	134 (40.0%)		
10+	825 (21.5%)	63 (15.4%)		
Number of deployments			5.23	0.073
0	2475 (65.4%)	235 (67.5%)		
1	756 (20.9%)	57 (16.5%)		
2+	452 (13.7%)	47 (16.0%)		
Branch of military			13.60	0.009
Army	1425 (46.4%)	135 (52.8%)		
Navy	800 (20.5%)	75 (17.1%)		
Air Force	890 (19.4%)	63 (13.7%)		
Marine Corps	229 (5.7%)	29 (6.7%)		
Other	364 (8.0%)	41 (9.6%)		
War-era served			41.49	<0.001
Vietnam	725 (43.3%)	40 (19.3%)		
Iraq/Afghanistan	214 (30.2%)	35 (46.4%)		
Persian Gulf	137 (12.0%)	17 (13.6%)		
Korean	40 (3.9%)	2 (1.4%)		
WWII	15 (1.0%)	3 (3.6%)		
Other conflict	97 (9.6%)	13 (15.7%)		
Trauma characteristics				
ACES score	1.3 (1.7)	3.5 (2.7)	23.50	<0.001
Number of lifetime traumas	8.4 (8.0)	13.4 (11.0)	11.58	<0.001
Military sexual trauma	252 (6.5%)	68 (16.1%)	48.99	<0.001
Clinical and health characteristics				
Lifetime major depressive disorder	469 (13.8%)	141 (42.8%)	221.43	<0.001
Lifetime posttraumatic stress disorder	324 (9.8%)	123 (36.6%)	244.58	<0.001
Lifetime alcohol use disorder	1367 (38.7%)	211 (59.6%)	67.10	<0.001
Lifetime nicotine use disorder	570 (15.4%)	110 (33.7%)	86.35	<0.001
Lifetime drug use disorder	331 (9.9%)	122 (38.8%)	276.51	<0.001
Concussion/traumatic brain injury	179 (5.1%)	50 (12.0%)	33.42	<0.001
Number of medical conditions	2.9 (2.1)	3.3 (2.4)	3.49	<0.001
Personality characteristics				
Agreeableness	5.1 (1.2)	4.7 (1.3)	6.46	<0.001
Emotional stability	5.3 (1.3)	4.6 (1.6)	10.22	<0.001
Conscientiousness	5.8 (1.1)	5.2 (1.4)	8.91	<0.001
Openness to experiences	4.8 (1.2)	4.8 (1.3)	1.09	0.14
Extraversion	3.8 (1.5)	3.4 (1.6)	5.41	<0.001
Protective psychosocial characteristics				
Perceived resilience	39.3 (6.6)	36.7 (8.2)	7.52	<0.001
Secure attachment style	2710 (70.6%)	149 (37.6%)	184.21	<0.001
Dispositional optimism	5.1 (1.4)	4.3 (1.7)	11.57	<0.001

Table 3 shows results of multivariable logistic regression analyses examining the association between lifetime history of homelessness and suicidality, current probable psychiatric conditions, and current functioning. Relative to veterans without a lifetime history of homelessness, veterans who reported a history of homelessness were at elevated odds of lifetime suicide attempt, attempting suicide two or more times, past-year suicide ideation, and more frequent past-year suicide ideation. They also were at greater odds of screening positive for current probable major depressive, PTSD, generalized anxiety, and drug use disorders; and scored lower on measures of mental, physical, and cognitive functioning, and higher on a measure of current psychosocial difficulties.

**Table 3.** Bivariate and multivariable analyses comparing rates of suicidality, current psychiatric disorders, and functioning by a history of homelessness

	No history of homelessness *N* = 3708 weighted 89.8% *N* (weighted %)	History of homelessness *N* = 343 weighted 10.2% *N* (weighted %)	Bivariate analysis χ^2^, *p*	Multivariable AOR
Suicidal behavior				
Lifetime suicide attempt	82 (2.4%)	54 (16.1%)	213.47, <0.001	2.28 (1.49–3.48)***
Number of attempts			214.46, <0.001	
0	3604 (97.6%)	279 (83.9%)		Ref.
1	52 (1.4%)	19 (5.2%)		1.89 (1.01–3.54)*
2 or more	29 (1.0%)	35 (10.9%)		3.11 (1.81–5.29)***
Past-year suicidal ideation	313 (9.9%)	72 (28.8%)	123.63, <0.001	1.39 (1.02–1.89)*
Frequency of past year SI			130.98, <0.001	
0	3395 (90.1%)	271 (71.2%)		Ref.
1	174 (5.1%)	31 (11.5%)		1.30 (0.87–1.95)
2	77 (2.4%)	19 (8.1%)		2.06 (1.26–3.37)**
3 or more	62 (2.3%)	22 (9.2%)		1.87 (1.14–3.06)*
Current disorders				
Major depressive disorder	215 (6.8%)	76 (26.0%)	168.86, <0.001	2.08 (1.53–2.83)***
Posttraumatic stress disorder	150 (4.6%)	69 (23.9%)	223.85, <0.001	2.42 (1.72–3.39)***
Generalized anxiety disorder	175 (6.3%)	57 (22.2%)	126.31, <0.001	1.72 (1.23–2.39)**
Alcohol use disorder	316 (9.9%)	44 (16.1%)	15.28, <0.001	1.06 (0.76–1.48)
Drug use disorder	239 (7.5%)	75 (28.2%)	169.89, <0.001	2.06 (1.52–2.79)***
Current functioning				Cohen *d* (95% CI)
Mental health functioning	50.9 (0.3)	48.7 (0.5)	26.26, <0.001	0.13 (0.02–0.23)
Physical health functioning	46.6 (0.4)	43.5 (0.6)	36.06, <0.001	0.14 (0.03–0.24)
Cognitive functioning	87.5 (0.6)	83.6 (0.9)	23.19, <0.001	0.11 (0.01–0.22)
Psychosocial difficulties	13.0 (0.6)	18.1 (1.0)	38.38, <0.001	0.15 (0.04–0.25)

AOR, adjusted odds ratio; 95% CI, 95% confidence interval.

*Note*. Weighted prevalence estimates are within the NHRVS subsample of veterans with and without a history of lifetime homelessness.

Significant association between homelessness and dependent variable: *p < 0.01; **p < 0.01; ***p < 0.001. Odds ratios and means are adjusted for age, sex, race/ethnicity, education, marital/partnered status, household income, enlistment status, years of military service, a branch of service, ACES, lifetime traumas, and military sexual trauma. Analyses of suicidality and self-harm, mental health treatment, and functioning variables are additionally adjusted for lifetime major depressive, posttraumatic stress, and alcohol, drug, and nicotine use disorders.

**Fig. 1.**
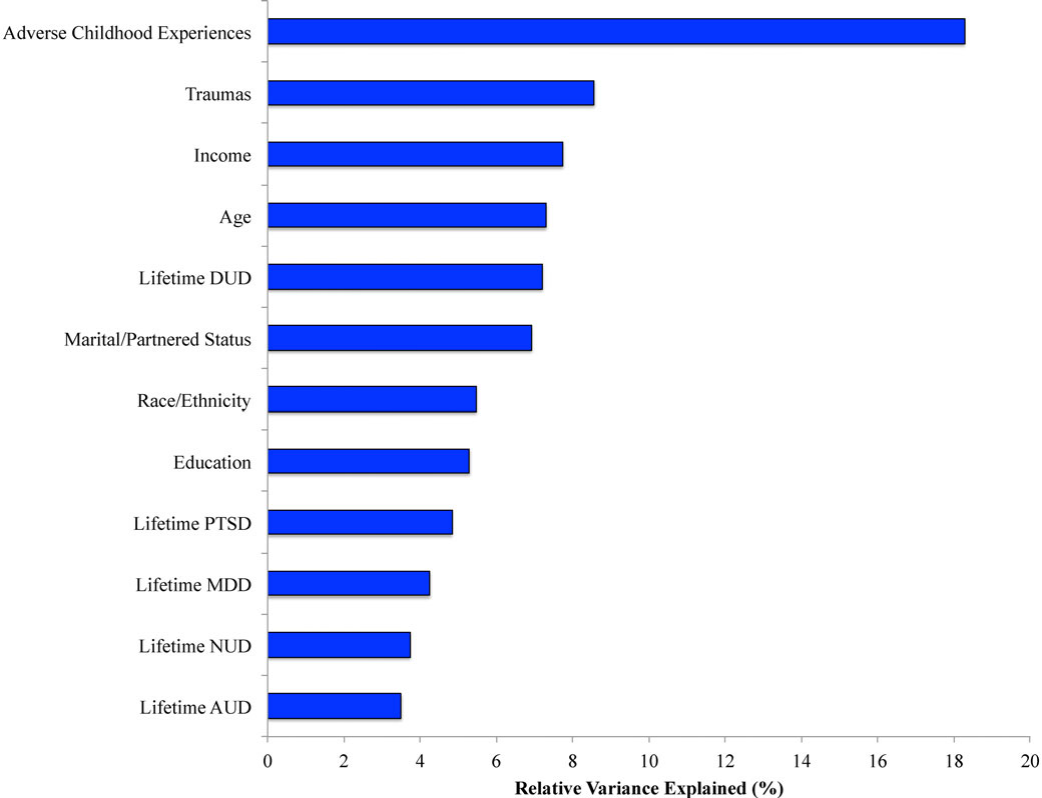
Relative importance of sociodemographic, military, trauma, clinical, personality, and protective psychosocial correlates of homelessness.

Results of relative importance analysis (Fig. 1) revealed that the majority of the relative explained variance (RVE) in lifetime homelessness was accounted for by a greater number of ACEs (18.3%), greater lifetime trauma burden (8.6%), lower current household income (7.7%), younger current age (7.3%), lifetime drug use disorder (7.2%), race/ethnicity (5.5%), and lower educational attainment (5.3%). Post-hoc analyses indicated that the following ACES were independently associated with homelessness: ‘Before your 18th birthday, was a biological parent ever lost to you through divorce, abandonment, or other reason?’ (4.6% RVE), ‘Before your 18th birthday, did an adult or person at least five years older than you ever touch or fondle you or have you touch their body in a sexual way? or attempt or actually have oral, anal, or vaginal intercourse with you?’ (4.2% RVE), ‘Before your 18th birthday, did a parent or other adult in the household often or very often swear at you, insult you, put you down, or humiliate you?’ (3.9% RVE), and ‘Before your 18th birthday, did you often or very often feel that you didn't have enough to eat, had to wear dirty clothes, and had no one to protect you? or your parents were too drunk or high to take care of you or take you to the doctor if you needed it?’ (3.8% RVE).

## Discussion

This study of a contemporary, nationally representative sample of U.S. military veterans found that the lifetime prevalence of homelessness was 10.2% (95% CI 9.3–11.2%), which is broadly consistent with prior national population-based studies of veterans (Edens et al., 2011; Holliday et al., 2021; Tsai & Cao, 2019; Tsai et al., 2016). For example, in an independent, nationally representative study that used a parallel sampling methodology as the current study, Tsai et al. (2016) found that the lifetime prevalence of self-reported homelessness among veterans was 8.5% (95% CI 7.1–9.9%). Approximately four-of-five veterans (82.4%) reported that their first episode of homelessness occurred following military service, an average of 10.6 years following military discharge until onset (s.d. = 12.6). These results accord with prior literature demonstrating a sleeper effect (e.g. delayed homelessness) following military discharge (Tsai et al., 2020). For instance, using two population-based samples of veterans, Tsai et al. (2020) found that the average time between discharge and the first episode of homelessness was 5.5 and 9.9 years, respectively. Furthermore, veterans with a history of homelessness in our sample reported an average of 15.8 months spent homeless. Collectively, findings suggest that one in ten veterans in the United States has experienced homelessness at some point during their lifetimes, with these individuals spending an average of nearly 1.5 years without stable housing.

ACEs emerged as the strongest correlate of lifetime homelessness, accounting for nearly 20% of the explained variance. This finding accords with prior literature from the general population, which has found that ACEs such as childhood physical and sexual abuse, neglect, and household dysfunction are independently linked with increased likelihood of homelessness during adulthood, even after controlling for sociodemographic, psychiatric, and substance use characteristics (Liu et al., 2021). Post-hoc analyses revealed that four types of ACEs accounted for the greatest proportion of explained variance in lifetime homelessness – loss of a parent during childhood (e.g. through abandonment or divorce), sexual abuse, emotional abuse, and neglect. It is plausible that veterans who reported greater ACEs may have been more likely to experience homelessness while in childhood (e.g. due to familial or economic instability), during adulthood following military service, or both (Metraux, Cusack, Byrne, Hunt-Johnson, & True, 2017; Montgomery, Cutuli, Evans-Chase, Treglia, & Culhane, 2013a; Tsai, Edens, & Rosenheck, 2011). For example, a prior analysis of homeless veterans found that over half reported childhood family instability, 40% reported a history of childhood abuse, and a third reported conduct disorder behaviors during childhood (Tsai & Rosenheck, 2013).

On average, veterans who reported a history of homelessness reported nearly triple the number of ACEs (mean = 3.5, s.d. = 2.7) relative to veterans who had never been experienced homelessness (mean = 1.3, s.d. = 1.7). These findings parallel results from a recent meta-analysis by Liu et al. (2021), who found that ACEs were nearly universal among adults in the general population experiencing homelessness, with 89.8% reporting one ACE and 53.9% reporting four or more ACEs. By comparison, this meta-analysis found that 3–5% of adults globally reported four or more ACEs. It is notable that our investigation found that ACEs emerged as the strongest factor independently associated with lifetime homelessness, even when considering other sociodemographic (e.g. race/ethnicity), psychiatric (e.g. lifetime PTSD, alcohol/drug use disorder history), and physical health characteristics (e.g. a number of physical health conditions). However, an important limitation is that the current investigation did not assess for serious mental illness (e.g. bipolar, schizophrenia) or personality disorders (e.g. borderline personality disorder), which have been identified as robust risk factors for homelessness among veterans (Hamilton, Suchting, Thomas, & Buck, 2021; Metraux et al., 2013; Tsai & Rosenheck, 2015). Together, these findings may have implications for homelessness prevention efforts, which are currently a top federal priority of the Biden-Harris administration (HUD, 2021c). There have been increased efforts to improve outreach, coordinated entry into homeless service systems, and addressing housing supply issues, which can help with secondary and tertiary prevention of homelessness. However, as this study shows, primary prevention of homelessness may require early-life interventions, possibly beginning as early as childhood. Furthermore, given that a substantial proportion of homeless veterans have dependent children (Tsai, Rosenheck, Kasprow, & Kane, 2015), helping children of homeless veterans may also be another form of primary prevention to help interrupt generational cycles of homelessness.

Cumulative trauma burden emerged as the next strongest correlate associated with homelessness, accounting for nearly 10% of the explained variance. Previous research has identified several processes by which combat and other types of trauma exposure may be related to increased risk for homelessness among veterans, although the directionality between trauma and homelessness are unclear (Fargo et al., 2012; Metraux et al., 2017; Montgomery, Fargo, Byrne, Kane, & Culhane, 2013b). Trauma exposure may indirectly increase the risk for homelessness through heightened financial strain caused by deteriorations in mental and physical health (Roos et al., 2013), which are established risk factors for homelessness (Elbogen, Lanier, Wagner, & Tsai, 2021; Tsai & Rosenheck, 2015). Of note, veterans who reported a history of homelessness were more than twice as likely to report a history of military sexual trauma (MST; 16.1%) relative to those without such histories (6.5%). This finding aligns with prior literature suggesting that MST may be an independent risk factor for postdeployment homelessness (Brignone et al., 2016). Indeed, prior qualitative research suggests MST exposure may initiate a ‘cycle of despair,’ whereby sexual trauma during military service can lead to increased secrecy, psychiatric symptoms, and substance abuse, which in turn increases the risk for eventual homelessness (Hamilton, Poza, & Washington, 2011).

Paralleling other recent findings (Holliday et al., 2021; Tsai & Cao, 2019), rates of current and lifetime suicidal behavior were substantially elevated among veterans who had experienced homelessness relative to those who had not. After adjustment for sociodemographic, trauma, and psychiatric characteristics, veterans who had experienced homelessness had more than twice the odds of having attempted suicide and nearly three times the odds of attempting suicide two or more times. Moreover, homelessness was independently associated with nearly double the odds of having a higher frequency of past-year suicidal ideation. Several plausible explanations may underlie the strong associations observed between lifetime homelessness and suicidal behavior. First, homelessness and suicidal behaviors share several risk factors, including low socioeconomic status, serious mental illness, substance use disorders, low social support, and poor physical health (Tsai & Cao, 2019). Second, a history of homelessness may serve as a risk factor for suicidal behavior, given that the experience of being (or becoming) homeless is associated with chronic stress. Indeed, prior longitudinal research has found that older veterans experiencing homelessness were twice as likely to die by suicide relative to those who were not over an 11-year period (Schinka, Bossarte, Curtiss, Lapcevic, & Casey, 2016). Third, the relationship may be bidirectional, in which housing instability may lead to declines in mental health, which in turn may heighten the risk for suicidality (Tsai & Cao, 2019). Conversely, suicidal thoughts or behaviors may lead to impairment in psychosocial functioning, which may increase the risk for financial strain and housing instability. Taken together, these results suggest that veterans with a history of homelessness represent a unique subpopulation of veterans that are at particularly high risk for suicide, and additional research is needed to better understand the processes by which homelessness and suicide risk are connected.

Similarly, veterans with a history of lifetime homelessness had high rates of current probable mental health and substance use disorders relative to those without such histories. For instance, veterans who had had previously experienced homelessness had approximately twice the odds of screening positive for current probable PTSD, depression, generalized anxiety disorder, and drug use disorder, even after stringent adjustment for sociodemographic, military, and trauma characteristics. A similar pattern emerged on measures of current functioning, which showed that veterans with a history of homelessness evidenced lower mental, physical, and cognitive functioning, as well as elevated psychosocial difficulties, even after additionally adjusting for psychiatric and substance use disorder history. Past research has shown that mental health and substance use problems generally precede homelessness and serve as risk factors for housing instability (Tsai & Rosenheck, 2015), although it is unclear from our cross-sectional data whether psychiatric problems were a cause or consequence of prior homelessness. Nevertheless, given the high mental health and functional burden associated homelessness in U.S. veterans, our findings underscore the importance of evidence-based treatments for mental health and substance use disorders, as well as additional screening and monitoring for suicide risk, in this population.

While this study had several strengths, including utilizing a nationally representative sample of U.S. veterans who do and do not use VA healthcare, several limitations should be noted. First, this study relied on cross-sectional data, and therefore the directionality of the associations observed in our study cannot be ascertained. Future longitudinal research is needed to better understand the temporal relations between homelessness, mental health, and suicidality among veterans (Nichter, Hill, Norman, Haller, & Pietrzak, 2020b). Second, the NHRVS did not assess for serious mental illness or personality disorders, which have been identified as strong risk factors for homelessness in the veteran population (Hamilton et al., 2021; Metraux et al., 2013). Third, the lifetime prevalence of homelessness found in our study is likely an underestimate, given that the NHRVS did not survey veterans who were currently experiencing homelessness or residing in institutions (e.g. state hospitals, prisons). Similarly, given that KnowledgePanel^®^ drew from a nationally representative sample of veterans, the majority of which are older, there were relatively small numbers of combat veteran subgroups of more recent war eras (e.g. Iraq and Afghanistan) which we found to be at heightened risk for experiencing homelessness. Further research is needed to evaluate the prevalence, correlates, and burden of homelessness in these more vulnerable segments of the veteran population (Nichter, Hill, Norman, Haller, & Pietrzak, 2020).

These limitations notwithstanding, the current study provides contemporary, population-based data on the prevalence and correlates of homelessness in the U.S. veteran population. Results suggest that one in ten veterans in the U.S. has experienced homelessness, with the vast majority of these individuals becoming homeless for the first time a decade or more following military service. Our findings showing that early and cumulative lifetime trauma burden were the strongest factors associated with the risk of lifetime homelessness have several public health implications. First, our results suggest that the Department of Defense (DoD) should consider incorporating screening measures for ACEs and cumulative trauma exposure into pre-discharge procedures (e.g. separation physicals) as a means to identify service members who may be at heightened risk for poor mental health outcomes and homelessness following discharge from the military (Nichter, Hill, Norman, Haller, & Pietrzak, 2020a). Incorporating such screenings into the standard of care could provide an effective, time efficient, and low-cost method to identify and refer service members to additional support programs (e.g. DoD Transition Assistance Program) both inside and outside the VA healthcare system following military service. While the pathways in which ACEs may increase the risk for homelessness in adulthood are not well-understood, they may include a combination of intrapsychic, interpsychic, and systemic factors. Intrapsychic and interpsychic factors may require psychological and psychiatric treatment to resolve so that veterans can live healthy and productive lives long-term, while systemic factors such as lack of educational/employment opportunities may need to be addressed at a community or national level through DoD and VA.

Second, on a broader level, results suggest that leveraging interventions to reduce early childhood adversity may be a critical component to end the cycle of homelessness among veterans and other at-risk populations. Indeed, prior research has found that approximately 20% of male and 50% of female veterans with current housing instability have children in their custody, a statistic that is concerning given that unstably housed veterans are more likely to have psychiatric disorders and chronic medical conditions (Tsai et al., 2015), which have been linked to inadequate parental care, as well as childhood abuse and neglect. As veterans with children represent a growing proportion of the homeless veteran population, additional research is needed to determine how to best support the psychosocial, financial, and mental health needs of this population to mitigate future homelessness and prevent children with a history of adversity from experiencing further adversities, and adverse mental health outcomes associated with homelessness.
